# Food Safety Aspects as Potential Impediment to the Adoption of Plant‐Based Alternative Protein Products in Sub‐Saharan Africa

**DOI:** 10.1002/fsn3.70050

**Published:** 2025-03-22

**Authors:** Fredrick O. Ogutu, Gertrude Okiko, George Wanjala, Susan Luvitaa, Boniphase Oure, Frank Vriesekoop, Claire D. Munialo

**Affiliations:** ^1^ Food Technology Division Kenya Industrial Research and Development Institute Nairobi Kenya; ^2^ Harper Food Innovation Harper Adams University Newport UK

**Keywords:** alternative proteins, bacterial contamination, food Safety, mycotoxins, plant‐based foods, sub‐Saharan Africa

## Abstract

The emergence and use of alternative proteins that seem to provide a sustainable solution to feeding the growing human population going into the future continue to gain momentum. However, there is not much research work on the safety of foods formulated using alternative proteins such as those of plant origin, aka plant‐based foods. Therefore, this review discusses the safety issues of producing and processing plant‐based foods. Special attention is paid to sub‐Saharan Africa, where most of the impact of climate change is felt, resulting in poor crop yields and reduced ability for thriving livestock production to serve as food for human consumption. Thus, the adoption of alternative plant‐based foods would be a good strategy to combat issues such as poor nutrition status that continue to be a plight to this region. There are safety concerns that relate to the introduction of alternative plant‐based protein foods which need to be overcome for these foods to be adopted in many food systems. The following safety concerns pertaining to plant‐based food production and processing were identified in the literature and are discussed in this work; the presence of allergens (from ingredients like soy, gluten, and nuts), anti‐nutritional factors (such as saponins, alkaloids, and isoflavones), mycotoxins, potential contamination with pathogenic microorganisms (e.g., Salmonella spp., *E. coli* , Bacillus spp., Listeria spp., *Clostridium sporogenes* , and *Geobacillus stearothermophilus* ), and the existence of potential carcinogens formed during processing (e.g., polycyclic aromatic hydrocarbons, heterocyclic aromatic amines, and nitrosamines), among others. This review concludes by recommending a thorough risk assessment of plant‐based protein foods to ensure wider successful adoption and use of plant‐based alternative food and protein products in SSA.

## Graphical abstract

Plant‐based foods are viewed to be more sustainable. There are safety issues related to plant‐based foods that can impede their adoption in local cuisines.

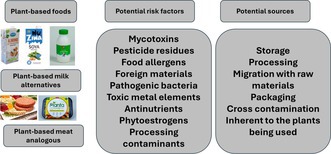

## 1 Introduction

The growing global population and changing dietary trends have increased the need for sustainable and nutritious food options, posing significant challenges for the food industry in developing effective alternative food sources (Newton and Blaustein‐Rejto 2021). It is expected that the world population will surpass 9.7 billion people by 2050, necessitating a paradigm shift in food production systems to address the issue of food insecurity and mitigate the environmental impact of conventional protein sources. One strategy that has been researched and suggested to enhance the availability of nutritious and healthy foods to feed the population that continues to grow is the shift to the use of alternative proteins from more sustainable sources, such as those of plant‐based proteins, which are generally viewed to be cheaper and readily available. These products are lauded for their reduced greenhouse gas emissions, reduced water use, and conservative land use compared to animal‐based proteins (Munialo et al. 2022; Munialo et al. 2014).

In sub‐Saharan Africa (SSA), where food insecurity, malnutrition, and the adverse effects of climate change are acute, plant‐based proteins present an opportunity to address these challenges sustainably. With a diverse range of indigenous crops (such as okra, Aizen, and soursop) and a long tradition of cereal (such as millet and sorghum), pulses (such as cowpea and mung beans), and vegetable‐based dishes (such as Bayenetu and Chakalaka), SSA is well‐suited for the incorporation of plant‐based diets in their food systems (Njeme et al. 2014). Indigenous crops are affordable and readily available resources for plant‐based protein production (Newton and Blaustein‐Rejto 2021; Talwar et al. 2024). Moreover, SSA's rich culinary traditions provide a favorable context for incorporating these protein‐rich ingredients into local diets and cuisines. However, the widespread adoption of plant‐based proteins in SSA faces a myriad of challenges including limited access to diverse plant‐based ingredients, cultural resistance to shifting dietary practices, and inadequate infrastructure for the processing and distribution of plant‐based foods (Hefferon et al. 2023; Talwar et al. 2024). Moreover, food safety concerns such as the risk of allergenicity, contamination by mycotoxins and bacterial pathogens, heavy metals and pesticide residue, and harmful compounds formed during food processing pose significant challenges to the SSA's food systems (Banach et al. 2023).

Safety concerns are of great significance when it comes to plant‐based foods due to an increase in consumption in recent years. Of critical significance are the substances whose presence should be negligible or below certain levels that could be considered safe for human consumption (He et al. 2020). Plant‐based foods have the potential of being contaminated by several risk factors that arise from the plant raw materials themselves to how the products are processed and how the final products are stored. The ingestion of these risk factors has the potential of causing serious harm and danger to human health, and this has a concomitant effect on the safe consumption of plant‐based foods. It is therefore vital to understand some of the potential risk factors that are associated with plant‐based foods for the related qualitative and quantitative methods of detection to be developed that have a high degree of accuracy and sensitivity. This is favorable and vital for guiding the optimization of the necessary conditions that are needed and used in the processing of these foods with the ultimate goal of ensuring that the consumer's health is protected while the establishment of relevant standards is promoted while ensuring healthy growth and development of the plant‐based food industry (Lin et al. 2023).

There is so much work that has been done, in particular in the developed countries, that pertains to the safety of plant‐based foods, including meat analogues. For instance, the presence of allergens in products that are made from soy has been highlighted and discussed in recent reviews by some authors (Alcorta et al. 2021; Munialo and Vriesekoop 2023; Präger et al. 2023). However, as the market for plant‐based foods continues to increase and spread across the globe to SSA, there is a need to research food safety‐related issues that would become an impediment to the adoption of these foods in local cuisines. Therefore, this review evaluated the presence of allergens, anti‐nutritional factors, mycotoxins, potential contamination with pathogenic microorganisms, and the existence of potential carcinogens formed during processing.

## 2 Methodology

There are a number of safety concerns that have been outlined in recent studies that highlight some issues that could impede the incorporation of plant‐based foods into the human diet. Therefore, there is a need for rigorous measures to mitigate the risks associated with alternative plant‐based protein production. These safety concerns in plant‐based foods include the presence of allergens and the potential for allergen cross‐contamination, the presence of antinutrients, bacteria, carcinogens such as heterocyclic aromatic amines that are thermally induced, contamination that can arise from inadequate food handling practices, the presence of mycotoxins, and other natural toxins and contaminants such as pesticides that are used during crop production (Hadi and Brightwell 2021). These factors are global food safety issues that have warranted the attention of many researchers.

To decipher some of the food safety issues that are associated with plant‐based foods, a scoping review was carried out. This work employed a scoping review methodology to explore the diverse and emerging literature on food safety concerns in plant‐based protein products, particularly within the context of SSA. The approach was chosen to map key safety risks, identify knowledge gaps, and synthesize peer‐reviewed and gray literature insights. A comprehensive search was performed using the databases (such as Scopus, PubMed, and Web of Science), alongside reports from organizations like the FAO and WHO. The search focused on studies published between 2014 and 2024, using keywords related to food safety (such as allergens, mycotoxins, bacterial contamination, and toxicants) and plant‐based proteins (such as soy, legumes, and cereals).

Inclusion criteria prioritized studies addressing safety risks in edible plant‐based protein products, emphasizing those relevant to SSA. Exclusion criteria filtered out works focused solely on nutritional aspects or non‐edible applications. Extracted data were grouped into key themes: presence of pathogens, allergenicity, presence of mycotoxins, bacterial contamination, and processing‐induced toxicants. Findings are synthesized into actionable insights, and tabulated summaries are created to present data on contaminant levels, associated health risks, and regional challenges. This scoping review provides a comprehensive and flexible framework for addressing the safety challenges of plant‐based proteins and informing their adoption in SSA.

## 3 Key Safety Concerns

There are several safety concerns that are associated with plant‐based foods. These concerns will be discussed in subsequent sections.

### 3.1 Presence of Pathogens

Plant‐based protein foods are not exempt from safety concerns related to foodborne pathogens, e.g., Salmonella spp., Bacillus spp., *E. coli* , Listeria spp., *Clostridium sporogenes* , and *Geobacillus stearothermophilus* (Kyrylenko et al. 2023). Contamination can occur during various stages of production, including farming, processing, and packaging. Inadequate food handling practices further exacerbate the risk of pathogen contamination and recontamination (Hadi and Brightwell 2021; Liu, Aimutis, et al. 2024). Therefore, ensuring that the safety and quality of the foods are maintained and ensuring an optimal hazard analysis and critical control points systems is crucial from farm to fork. It is also important to ensure a consistent supply of raw materials, as this could affect the downstream processes and product hurdle system for maintaining the shelf life of the final products.

In comparison to the well‐known food safety issues like microbial growth and inactivation kinetics in traditional meats and poultry, the risks of plant‐based meat alternatives (PBMAs) are largely unknown, mainly because of the rapid rise and popularity among consumers (Kabisch et al. 2024). There are mixed views regarding the presence of pathogens in plant‐based foods. Some authors suggest that plant‐based alternative products have a relatively lower microbial load than traditional animal proteins (Fu et al. 2023; Liu et al. 2023; Malila et al. 2024). However, the intrinsic properties of these foods are shown to be characterized by a pH of 6–7 and a water activity of more than 0.98, with a high protein and moisture content, making them ideal candidates for both microbial and enzymatic spoilage (Tóth et al. 2021). Other authors have suggested the addition of ingredients that are grain‐based, soy protein, and pea proteins, as well as food starches to plant‐based products such as plant‐based meats, which does introduce new pathogens in addition to quality risks attributed to the presence of pathogenic bacteria such as *Bacillus cereus* and lactic acid bacteria (Geeraerts et al. 2020) that cause a deterioration in the shelf‐life and keeping quality of these products. One may argue that the processing conditions used in the formulation of plant‐based foods that require the application of high temperatures, such as extrusion, can result in some of the pathogens being killed. However, some of these methods are still in the infancy stages in SSA, due to the high costs needed to install the extruders in addition to the technical know‐how needed to operate them (Egal and Oldewage‐Theron 2020). As such, these techniques are yet to be fully operational in most food industries in SSA and a failure in the processing or production can result in some of these pathogens making their way into the vulnerable food systems undetected, posing a major health risk to consumers.

Some studies show that various microbes can be introduced during harvesting, storage, or manufacturing, heightening the risk of microbial contamination (Kyrylenko et al. 2023). Raw materials for plant‐based proteins are often sourced close to the soil, potentially exposing them to many contaminants, including spore‐forming bacteria capable of surviving heat treatments. However, some authors have reported that the protein raw materials that are appropriate for the formulation of meat analogues do not pose microbial food safety risks (Dušková et al. 2024; Liu et al. 2023; Tóth et al. 2021). Nonetheless, the meals that included the use of meat analogues in their preparation proved to be more susceptible to quality deterioration and became more perishable due to bacterial contamination (Tóth et al. 2021). Some research indicates a slightly elevated food safety risk associated with meals prepared using plant‐based meat analogues compared to their animal‐origin meat counterparts as aforementioned. For instance, in a storage experiment, lower contaminants by microbes were observed in raw protein sources, yet meals containing plant‐based meat analogues showed faster microbial proliferation, particularly when not adequately cooled down (Tóth et al. 2021). Consequently, plant‐based meat analogues could inadvertently foster the growth and survival of pathogenic and spoilage microorganisms. This necessitates preventative controls to ensure microbial quality and safety. Therefore, to be able to combat this issue, an effective and robust food safety management system that includes traceability and routine product testing should be operated to support the formulation and production of meat analogues.

The other pathogen‐related safety issue that can impede the use of plant‐based foods is microbial contamination, encompassing bacteria and molds. Bacteria can either encompass pathogens that are infectious or strains that can produce toxins, both of which pose significant safety concerns within the field of food microbiology. The inactivation of bacteria usually occurs during food processing operations that include cooking and extrusion. Some studies have however shown that some bacteria that have the potential of forming endospores (such as *Clostridium* spp. or *Bacillus* spp.) and other bacteria (such as *Enterococcus faecium* and *Lactobacillus sakei* ) can outlive the regime that is used to heat/cook the product or can still be present to some extent in the final product as a result of post‐extrusion process re‐contamination (Kyrylenko et al. 2023) which would mainly occur when food products are processed under suboptimal conditions or stored improperly. While the thermoplastic extrusion process utilized in meat analogues production has been shown to effectively deactivate spores (Hadi and Brightwell 2021), there remains apprehension regarding the survival and germination of spore‐forming bacteria like *Bacillus cereus* or *Clostridium perfringens* during this process. A study by Kabisch et al. (2024) isolated various *Clostridium* species in 7 out of 10 meat analogue products tested, highlighting the persistence of microbial risks despite processing (Kabisch et al. 2024). Adherence to good manufacturing practices, stringent hygiene protocols, and appropriate storage conditions is imperative for mitigating microbial contamination and upholding the microbiological safety of plant‐based protein products (Kyrylenko et al. 2023; Roch et al. 2024). By implementing robust preventive measures, stakeholders can minimize microbial risks and enhance the safety and quality of plant‐based meat analogues, thereby bolstering consumer confidence in these innovative products that continue to make their way to SSA markets.

### 3.2 Allergenicity

Many plant proteins, including legumes (such as lupine, soy, and pea) and cereals (such as wheat and barley) proteins among others are known allergens (Hadi and Brightwell 2021; Rizzolo‐Brime et al. 2023). Individuals who are at risk of wheat allergies and coeliac disease may also be susceptible to an adverse allergic reaction that may occur following the ingestion of gluten, which is a protein that is found in grains, such as barley, rye, and wheat (Daly et al. 2020). Food allergens have the potential of enacting an immune response, and the symptoms could end up being catastrophic due to the elevated risks of anaphylaxis shock (Cianferoni and Muraro 2012). The severity of allergic reactions that are food‐induced is in most cases impacted by the type of allergen as well as the dose of the product consumed (Newton and Blaustein‐Rejto 2021; Urugo 2023). It is worth noting that as levels of people with allergies increase globally, SSA is not left out in this increase with some authors suggesting that allergies and allergic diseases are no longer “a rare fact” in Africa (Mvoundza Ndjindji and Djoba Siawaya 2022). Therefore, there is a need for plant‐based foods to be mapped for the presence of allergens and this information needs to be clearly communicated to consumers, especially in cases of known allergic reactions, as this would help such individuals when it comes to making informed food choices. This is particularly the case given that most of the plant‐based proteins that are used in the formulation and production of plant‐based foods are soy, and peanuts, which are common food allergens.

There is also the possibility that increased consumption of products that contain considerable amounts of proteins of plant origin, such as soy, lupine, wheat, and pea, among others, could provoke the body's immune system and trigger an allergic response in individuals who have not previously experienced any issues with these foods (ILSI 2010; Rizzolo‐Brime et al. 2023; Vissamsetti et al. 2024; Newton and Blaustein‐Rejto 2021; Urugo 2023). A summary of common allergenic contaminants in plant‐based proteins and associated risks is provided in Table 1. One major issue that has resulted in allergenic contamination in SSA is the labeling of food products. Proper labeling and reading of food labels is essential but, in most cases, difficult to achieve in most countries in SSA which is mainly attributed to inadequate labeling legislation in some of the countries in addition to high levels of illiteracy in some of the geographic locations in SSA (Hossny et al. 2019). Therefore, to minimize the unintentional contamination of food products with allergens, governmental authorities as well as the food industries in SSA should work on implementing food allergen labeling.

**TABLE 1:** Common allergenic contaminants in plant‐based proteins and associated risks.

Contaminant	Source	Health risks	Regional context in SSA	References
Soy protein (prolamins, cupins, Glycinin m 3 and Gly m 4)	Soybeans and soy‐based products	IgE‐mediated immunological reactions may manifest as cutaneous, gastrointestinal, cardiovascular, and respiratory symptoms.	Increasing use of processed plant‐based products without clear allergen labeling.	Hadi and Brightwell (2021)
Gluten	Wheat and wheat derivatives	Gluten intolerance and celiac disease; potential cross‐reactivity with other grains	Limited awareness of gluten intolerance and lack of gluten‐free product availability. The HLA‐DQ2 gene, associated with coeliac disease, is less prevalent in sub‐Saharan African populations than in Northern Africa and Europe.	Lionetti et al. (2014)
Peanut protein Ara h 1–2‐3, cupin storage proteins.	Peanuts and peanut‐based products	Severe allergic reactions, including anaphylaxis; cross‐reactivity with lupine and pea proteins	Peanut allergy is perceived to be underdiagnosed in Africa. Severe allergic reactions to peanuts are rare despite frequent consumption and sensitisation, and peanuts are widely used in local diets.	El‐Gamal et al. (2017), Bublin et al. (2019)
Lupine protein	Lupine flour and derivatives	Allergic reactions, particularly in individuals allergic to peanuts due to cross‐reactivity	Increasing introduction of lupine as a soy alternative; low consumer awareness	Newton and Blaustein‐Rejto (2021)
Pea protein	Peas and pea‐based products	Cross‐reactivity with peanut allergens; potential immune responses in sensitive individuals	Growing use in meat analogues and plant‐based formulations; limited allergen labeling	Kabisch et al. (2024)

Pea, among other plant proteins, has continued to be used in the formulation of many plant‐based foods. Pea in the form of protein concentrates and protein isolates can be incorporated in various plant‐based foods for the purposes of adding bulk and increasing protein content levels. However, there is a possibility that this could potentially lead to consumption‐induced allergic reactions following the consumption of these products. Post‐consumption allergic symptoms have been reported from individuals following the consumption of peanut protein, suggesting the possibility of cross‐reactions owing to the similarity between peas and peanuts (Baune et al. 2022). An increased exposure to pea protein as a result of ingesting plant‐based foods that are made with pea protein could result in a more frequently encountered allergenic food. Therefore, there is a need for the potential risks that surround the incorporation of proteins of plant origins in food production to be addressed and possible ways, in particular, of minimizing or eliminating allergenicity of these proteins to be researched and incorporated in food processing. Addressing the potential risks of these allergens, reducing the cost of production, and improving organoleptic properties could ultimately enhance the safety and trustworthiness of plant protein‐based food products in SSA.

### 3.3 The Presence of Antinutrients in Plant‐Based Foods

Plant‐based foods often have anti‐nutritional factors like saponins, alkaloids, isoflavones, phytosterols, alkaloids, and saponins, with some having hydrogen cyanide (Ogutu et al. 2018). Most plant foods, nuts, legumes, vegetables, and cereals contain anti‐nutrient compounds. For instance, some plant‐based ingredients, such as legumes, have been shown to contain antinutrients such as lectins, goitrogens, protease inhibitors, phytic acid, phytoestrogens, phytates, oxalates, saponins, and tannins that can have an adverse effect on human health (Bogueva and McClements 2023; Ogutu et al. 2018). Some of the common antinutrients that are present in plant‐based sources of food are listed in Table 2.

**TABLE 2:** Some of the common antinutrients that are present in different plant‐based foods. This information has been adapted from the work of (Munialo 2024a).

Source	Amount	Type
Grains such as barley, corn, millet, Kamut, oat, spelt, sorghum rye, and wheat	50‐74 mg/g 35–270 mg/100 g	Phytic acid Oxalates
Legumes such as chickpeas, lentils, peanuts, and soya beans	2–200 mg/100 g 106–170 mg/100 g 1.8–18 mg/g 6.7 mg/100 g 8 mg/kg 386–714 mg/100 g	Cyanide Saponins Tannins Trypsin inhibitor Oxalates Phytic acid
Nuts for example almonds, cashew nuts, Brazil nuts, hazelnuts, macadamia nuts, pignoli, pistachio, and walnuts	150–9400 mg/100 g 37–144 μg/g 40–490 mg/100 g	Phytic acid Lectins Oxalates
Nightshades such as eggplant, tomato pepper, and potato	0.82–4.48 mg/100 g 0.19 mg/100 g 0.16–0.25 mg/100 g 1.6–10.5 mg/100 g	Phytic acid Tannins Saponins Cyanide
Pseudo‐grains such as amaranth, wheat, buckwheat, quinoa, and Teff	0.04–2.14 ppm 0.5–7.3 g/100 g	Goitrogens Lectins Phytic acid Saponins
Seeds such as flaxseed, sesame seeds, sunflower seeds, poppy seeds, and pumpkin seeds	0.251 mg/mL 140–370 ppm 1–10.7 g/100 g	Alpha‐amylase inhibitor Cyanide Phytic acid
Tubers such as carrot, tapioca (or manioc), sweet potato, Jerusalem artichoke, and yam	0.4–2.3 mg/100 g 4.18–6.72 mg/100 g 0.06–0.08 mg/100 g	Oxalates Tannins Phytates

Common examples of natural toxins that are found in plants include lectins which are found in green, red, and white kidney beans; cyanogenic glycosides which are found in bitter apricot seeds, bamboo shoots, cassava, as well as in flaxseeds; glycoalkaloids which are found within potatoes; 4 colchicine which are often found in fresh lily flowers; and muscarine which are commonly found in some wild mushrooms varieties (Dixit et al. 2011; Hajšlová et al. 2004). Hence, it is vital that all ingredients that are derived from plants and used in product formulation undergo thorough and meticulous selection and processing procedures with the ultimate aim of preventing, eliminating, or deactivating and neutralizing these toxins.

There are factors in plant‐based proteins that have the ability to decrease the bioavailability of nutrients, which are resistant to proteolysis due to structure, certain conformations of proteins, and the presence of antinutrients (Banach et al. 2023). Lectins are of concern when it comes to the formulation of soybeans and rice milk substitutes. Soy‐based meats and milk analogues may contain soy isoflavone compounds, for example, phytoestrogens, which have been linked to several health concerns (Liu, Aimutis, et al. 2024). Some researchers reported that excessive consumption of phytoestrogens may provoke adverse health effects on individuals' reproductive health (Bogueva and McClements 2023). Soy is the key ingredient used in the formulation of most plant‐based meat alternatives (PBMAs) and alternatives to dairy milk. However, a higher intake of soy foods and soy isoflavones has been associated with a reduction in sperm concentration (Chavarro et al. 2008), and this becomes of great concern as to the impact of the consumption of soy‐based plant foods on fertility given that the level of fertility in SSA is projected to fall from 3.1 births per woman that were reported in 2010 to 2.1 births in 2050 (Tesfa et al. 2023). Soybeans also contain ~2% soy saponins, alkaloids, tannins, phlobatannins, oxalate or cyanogenic acid, and anthraquinones (Dixit et al. 2011). Additionally, free phenolics that are found in leguminous vegetables such as peas and beans and cereal grains such as wheat and rye are known anti‐nutritional factors that affect the bioavailability of proteins, some minor minerals such as Zn, Fe, and Cu, as well as some major minerals such as Ca and P (Samtiya et al. 2020), and this has the potential of heightening nutrient deficiencies in a continent that is already plagued by malnutrition. Various cooking regimes reduce the levels of anti‐nutritional factors (Gupta et al. 2013; Hendek Ertop and Bektaş 2018). Additionally, in the processing of PBMAs, carrageenan is used as a binder and gelling agent, but it chelates minerals, leading to reduced mineral bioavailability and protein absorption of foods thanks to their chelating properties (Mittal and Bhuiyan 2023; Zhang et al. 2022), thus leading to micronutrient malnutrition and mineral deficiencies, which is a common occurrence in SSA (Ecker et al. 2024; Othoo et al. 2021).

### 3.4 The Potential Risk of Mycotoxins

Mycotoxins, which are hazardous toxic secondary metabolites produced by a variety of fungal species, represent a critical concern in food safety due to their mutagenic, teratogenic, and carcinogenic properties (Jallow et al. 2021). Among the most prevalent mycotoxins that are found in food commodities are aflatoxins and ochratoxins from *Aspergillus* species, ochratoxins and patulin synthesized by *Penicillium*. Additionally, *Fusarium* species have been shown to generate fumonisins, deoxynivalenol (DON), and zearalenone (Ekwomadu et al. 2021). Globally, mycotoxins, such as aflatoxins, fumonisins, patulin, and ochratoxins, among others, are shown to account for several acute and chronic illnesses in humans (Awuchi et al. 2022). Owing to a favorable climate that allows for the growth of fungi coupled with regulations that are inadequate, outbreaks of acute mycotoxicosis that end up resulting in deaths are a common occurrence in SSA (Kibugu et al. 2024). There have been reports of several agricultural crops that are grown in SSA and are considered important to health and livelihood being contaminated with mycotoxins (Atongbiik Achaglinkame et al. 2017; Ismail et al. 2019).

Mycotoxins also exhibit thermal stability, making them resistant to conventional food processing methods (Kutasi et al. 2021). Mycotoxins have been shown to be present in some of the raw ingredients that are often used in the formulation of PBMAs, such as soy (legumes), oats and rice (cereals), and almonds and walnuts (nuts). Mycotoxins, being “mutagenic, teratogenic, and carcinogenic”, are potent toxins that can result in harmful health effects in humans (Atongbiik Achaglinkame et al. 2017). The severity of mycotoxin toxicity is influenced by factors such as exposure time, concentration, and individual sensitivity (Bennett and Klich 2003; Mihalache et al. 2023). Notably, mycotoxins exhibit thermal stability within conventional food‐processing temperatures, posing challenges for complete destruction (Jallow et al. 2021; Kutasi et al. 2021). It is therefore important to institute a mycotoxin regulatory framework in plant‐based foods so as to minimize their contamination and health impact.

In PBMAs production, grains and legumes serve as primary ingredients, potentially introducing mycotoxins and plant alkaloids into the final products. Studies have identified the presence of aflatoxins (AFs), ochratoxin A (OTA), and trichothecenes in European legumes and PBMAs, with contamination levels that have been shown to range from 0.2 μg/kg Afs present in peas to 367.5 μg/kg deoxynivalenol (DON) present in soy‐based burgers (Mihalache et al. 2023). Additionally, OTA has been detected to be present in plant‐based foods and ingredients (Solfrizzo et al. 2015). Fumonisin FB1, which has predominantly been found in corn, soybeans, rice, and other commodities, presents a further concern (Bogueva and Mcclements 2023). Of concern is the lack of regulations regarding mycotoxin and plant alkaloid levels in commonly used legume‐based meat alternatives like soy, pea, and chickpea within the European Union (Mihalache et al. 2022). This regulatory gap, coupled with the rising consumption of PBMAs (Munialo 2024b), and global issues where plant‐based foods can be exported to SSA as part of trade or as food aid could contribute to an increased dietary intake of mycotoxins and plant alkaloids.

While transitioning to a plant‐based diet may eliminate certain cancer risk factors associated with processed meat consumption (Munialo and Vriesekoop 2023), inadequate regulation of mycotoxins in PBMAs could introduce unforeseen health risks. The co‐occurrence of mycotoxins in soy‐based PBMAs, as demonstrated by (Mihalache et al. 2022), underscores the potential additive or synergistic toxic effects of mycotoxin mixtures, highlighting the need for policymakers to regulate mycotoxin levels in PBMAs to ensure consumer safety.

In tropical areas such as SSA, due to prevailing conditions of temperature and humidity, specific mycotoxins like fumonisin and aflatoxins are endemic and are in very high levels in some crops (Ankwasa et al. 2021; Omara et al. 2021). Most plant‐based protein sources in the region tend to be infected by aflatoxins. For instance, a recent review on global mycotoxin occurrences established higher occurrences in developing countries where regulatory limits do not exist in some cases or are not adequately enforced in cases where they exist. Moreover, the raw materials storage infrastructure cannot guarantee quality materials for long‐term storage. In Kenya, there have been many incidences of mycotoxin and aflatoxin intoxication, even leading to acute deaths, more so in corn; moreover, peanuts and other cereals and legumes are equally often contaminated by mycotoxins (Omara et al. 2021). In other East African countries including Burundi, Uganda, Tanzania, Rwanda, and South Sudan, mycotoxins and aflatoxin have widely been reported, with key ones being aflatoxins, fumonisins, zearalenones, and DON (Ankwasa et al. 2021; Kimanya 2015). The highest aflatoxin contamination levels that have been reported in maize and peanuts among the East African countries were 48,000 μg/kg and 7525 μg/kg, respectively, which is far above the acceptable level of 4 μg/kg to 10 μg/kg (Mutegi et al. 2009). These recorded values were in products from Kenya while the highest fumonisin contamination that was detected in maize was 18,184 μg/kg, which was found in products from Tanzania (Ankwasa et al. 2021). The 18,184 μg/kgfumonisin content detected in maize is several folds above globally accepted Fumonisin limits of 2–4 ppm (Azizi and Rouhi 2013).

Mycotoxin contamination in SSA is of significant concern. The warm and humid climate that covers most areas within the region offers ideal conditions for the growth of mycotoxin‐producing fungi as aforementioned (Nji et al. 2022). This, coupled with reliance on traditional farming methods and poor storage infrastructure, creates perfect conditions for fungal growth and contamination of the major staple crops such as maize, groundnuts, and sorghum (Udomkun et al. 2017). Limited awareness and enforcement of mycotoxin regulations further exacerbate the issue, increasing exposure risks among the region's vulnerable populations (James and Zikankuba 2018; Kimanya 2015). SSA is an emerging market for alternative plant‐based protein products. Mycotoxin contamination of protein sources derived from legumes, oilseeds, and grains can significantly compromise the safety and quality of the products. Failure to meet food safety standards may limit their market potential and hinder adoption as a sustainable protein alternative. Moreover, contaminated products have limited local and international marketability, undermining the region's food security and economic stability. This creates a double burden of health challenges and financial losses, particularly for smallholder farmers who rely on plant‐based proteins as a primary source of nutrition and income. Table 3 highlights contaminating mycotoxins in plant‐based proteins, their occurrence levels, and locations where they have been detected in various products that range from freshness to maize and peanut butter.

**TABLE 3:** A summary of some of the mycotoxins that have been reported in various products from locations in SSA.

Product	Mycotoxin(s)	Occurrence level (s) μg/kg	Location	Source
Fresh nuts	Total Aflatoxins	5.7–22,168	Ghana	Kortei et al. (2022)
	Aflatoxin B1	12.5–528.3	Benin and Togo	Egal et al. (2005)
	Aflatoxin B1	0–7525	Kenya	Mutegi et al. (2009); Omara et al. (2021)
	Aflatoxin B1	1.5–937	DR Congo	Gachara et al. (2024)
	Total Aflatoxins	15–11,900	Ethiopia	Gelaye (2024)
	Total Aflatoxins	7–500	Malawi	Matumba, Van Poucke, Monjerezi, et al. (2015)
Dry roasted nuts	Aflatoxin B1	5–165	Nigeria	Bankole et al. (2005)
	Aflatoxin B2	6 0–26		
	Aflatoxin G1	5–20		
	Aflatoxin G2	7–10		
Peanut butter	Total Aflatoxins	34.2–15.6	Malawi	Matumba, Van Poucke, Monjerezi, et al. (2015
Dried Maize kernels	Total Aflatoxins	22–190	Benin	Gnonlonfin et al. (2019)
	Aflatoxin B1	7.6–27.2	Benin, Togo	Aasa et al. (2023)
	Total Aflatoxins	5–20	Malawi	Probst et al. (2014)
		2–162	Sierra‐Leone	
		1–1407	Somalia	
		0–435	Uganda	
		0–87	Kenya (Rift Valley)	
		0.1–57	DR Congo (Bas)	
		0–122	Cameroon	
	Aflatoxin B1	3–1081	Tanzania	Kayanda et al. (2024)
	Fumonisin B1	16–18,184		
	Fumonisin B2	178–38,217		
	Total Aflatoxins	> 20	Uganda	Atukwase et al. (2024)
	Total Aflatoxins	4–1400	Nigeria, Ghana	Perrone et al. (2014)
Beer	Total Aflatoxins	0–185	Malawi	Matumba, Van Poucke, Njumbe Ediage, et al. (2015)
	Total Fumonisins	493–3303		
Flour	Aflatoxin B1	0.32–1.64	Congo	Manjula et al. (2009)
	Aflatoxin B1	2.7	Tanzania	Manjula et al. (2009)
	Fumonisin B1	29.8		
	Ochratoxin A	1.9		
Chips	Total Aflatoxins	5.2–14.5	Cameroon	Monono et al. (2024)
	Aflatoxin B1	0.4–4.38	Congo	Manjula et al. (2009)
	Aflatoxin B1	0–33.8	Tanzania	Manjula et al. (2009)

### 3.5 Other Contaminants That Could Potentially Be Present in Plant‐Based Foods

The processing of foods plays a significant role in the presence of contaminants and toxicants. Food processing can either increase or decrease the functional characteristics, nutritional aspects, digestibility, and bioavailability of particular ingredients and foods (Rizzolo‐Brime et al. 2023). For example, there are a number of carcinogens, e.g., heterocyclic aromatic amines, nitrosamines, and polycyclic aromatic hydrocarbons, which could be produced during thermal processing of foods (Lu et al. 2017). Other examples include the glycidyl fatty acid esters, the 2‐monochloropropanediol (2‐MCPD), and the 3‐monochloropropanediol (3‐MCPD) (Hadi and Brightwell 2021), all of which can impact the quality and safety of food and food products.

**FIGURE 1:**
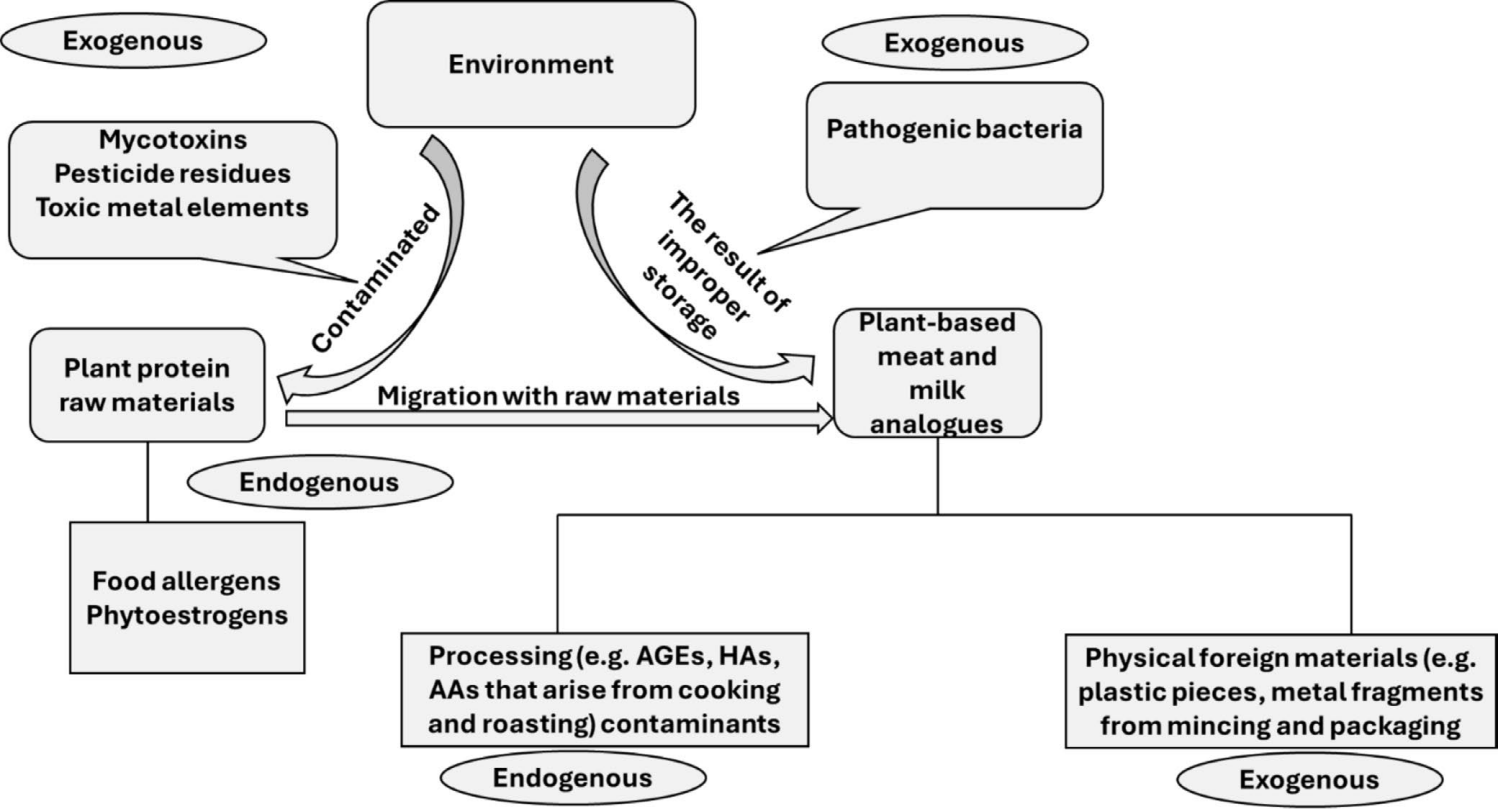
Possible sources of the potential food safety risk factors in plant‐based foods. Abbreviation: HAs: heterocyclic amines, AGEs: advanced glycation end products, and AAs: acrylamides. Adapted from Lin et al. (2023).

Some of the issues that impact the safety of food occur during the cultivation of crops; for instance, the use of pesticides during crop production can result in residual toxic effects when these chemicals make their way into the food chain. A possible source of potential food safety risk factors that are associated with plant‐based foods is summarized in Figure 1.

The utilization of lipid sources that contain trans‐fatty acids that are formed during partial hydrogenation of vegetable oils has been reported to also have the potential of enacting adverse effects on the healthiness of ultra‐processed foods (Bogueva and McClements 2023). The consumption of ultra‐processed food has been associated with many adverse health consequences linked to various comorbidities like types of cancers, cardiovascular diseases, type 2 diabetes, and obesity, and all‐cause mortality (Rizzolo‐Brime et al. 2023). A study on the small intestine and colon cancer promotion effect revealed a carcinogenic potential of ultra‐processed foods. This carcinogenic potential was found not only in commercially available processed meat products but also in a processed vegan product in the A/J Min/+ mice model (Bedale et al. 2023). Moreover, depending on the degree of processing, PBMAs were shown to have the potential of containing a higher diversity of other ingredients needed that are added with the aim of mimicking the characteristics as well as the sensorial attributes of conventional products that are made using animal‐based proteins (Bedale et al. 2023) making PMBAs to be classified as ultra processed foods. All these provide grounds for an array of sources from where potential hazards can arise and develop. Therefore, a thorough risk assessment is required to ensure the safety of these products for human consumption (Mihalache et al. 2022).

The use of genetic engineering in the production of PBMAs can introduce potential safety risks such as the presence of viruses or infectious prions, besides allergens (Banach et al. 2023). To reduce the potential risk of genetic engineering in meat analogues, it is crucial to implement strict regulations and thorough testing procedures. Firstly, comprehensive safety assessments to screen for viruses, infectious prions, and other potential hazards should be conducted on the genetic modifications used in PBMAs to ensure that they do not pose any health risks to consumers (Hadi and Brightwell 2021; Bedale et al. 2023). Moreover, clear labeling regulations should be put in place to ensure that consumers are fully informed about the ingredients and production methods used in plant‐based meat substitutes, including potential allergens such as soy, gluten, and nuts, to prevent any adverse reactions in individuals with allergies (Bahamon et al. 2023), as aforementioned. The other issue that impedes the incorporation of plant‐based proteins in food formulation is their production. This is mainly related to plants being used for genetic modification (Liu, Panda, et al. 2024) and have mainly been viewed with the concern of new allergy risks emanating from the genetically modified species. Africa, which is a developing continent that is faced with an increasing level of malnutrition, inadequate food production technologies, and food crises, has been reported to be slow in accepting and adopting genetically modified crops. This hesitancy has been shown to emanate from unfavorable policies that are shaped by public opinion, even though there could be the potential for these crops to achieve the zero‐hunger agenda (Ibrahim et al. 2022).

During the formulation of PBMAs and alternatives to dairy milk, it is common to incorporate additives that include antimicrobials, flavorings, colourings, binding agents, sweeteners, and preservatives (Ibrahim et al. 2022; Mihalache et al. 2022), which would extend the shelf life, among others. Food additives are reported to have the potential to trigger a slew of serious health issues. As such, the consumption of PBMAs and milk that incorporate a mixture of numerous additives in their formulation could have an impact on health. There is a need, therefore, to ensure that good manufacturing practices are observed, which would limit the impact of the additives on human health.

Governments and regulatory bodies in SSA need to establish robust food safety regulations and standards to respond to the needed consumer protection (Tachie et al. 2023; Tyndall et al. 2024). It is imperative to establish strict food handling practices and production standards to prevent contamination and ensure the overall safety of plant‐based meat substitutes. This could involve implementing guidelines for the separation of allergen‐containing ingredients during production and establishing best practices for cleaning and sanitization to minimize the risk of contamination (Alcorta et al. 2021).

## 4 Some of the Other Challenges That Increase the Food Safety Risks Associated With Plant‐Based Alternative Foods

Food labels originally came into existence with the goals of facilitating trade between countries and ensuring the overall safety of foods. Nowadays, expectations have changed dramatically with the realization that food labels have the potential to serve as an educational and informational tool (Todd et al. 2021). However, the effective use of food labels in some African countries has been shown to have its challenges. For example, several factors were reported to impair the effective use of the labels in South Africa. These include (i) practical barriers that impact the label use; (ii) some contextual and personal variables that influence the engagement with label information; (iii) some messaging preferences (for instance, for claims that are positively worded compared to more cautionary statements); (iv) some complexities that involve stakeholders and this was mainly related to trust and responsibility; and (v) ambassadors to change (Todd et al. 2021). There have also been reports of the lack of clear labeling regulations in other African countries when it comes to things like foods that have been genetically modified (Oh and Ezezika 2014). The lack of clear labeling regulations further compounds these challenges, potentially leading to confusion and undermining consumer trust in plant‐based products (Shireen and Wright 2024). Allergens like soy, gluten, and nuts are often used in plant‐based meat substitutes, posing a risk to individuals with allergies if not properly labeled or handled (Hadi and Brightwell 2021; Ibrahim et al. 2022; Mihalache et al. 2022; Tyndall et al. 2024).

## 5 Conclusions

The adoption of alternative foods, and in particular plant‐based foods, is suggested to be an excellent option to combat global warming issues that are related to livestock production. As such, these foods are viewed to be more sustainable and could contribute toward the UN Sustainable Development Goals. However, the adoption of plant‐based foods in particular in SSA could be impeded by the myriad of safety‐related issues such as allergenicity, presence of mycotoxins, and bacterial contamination, among others, which would need to be overcome to pave the way for the adoption of these foods into local cuisines and diets. Contaminated products have limited local and international marketability, which can undermine and exacerbate the region's food security crises and economic stability. There arises the need for various stakeholders to work together when it comes to the formulation, production, and distribution of plant‐based foods, and this provides opportunities for cross‐discipline collaborations to ensure the availability of sustainable and nutritious diets that are safe for human consumption in SSA. This will result in an increase in food security for SSA and the betterment of the livelihoods of the population that continues to increase and needs feeding using diverse yet safe food sources.

## Author Contributions

**Fredrick O. Ogutu:** formal analysis (equal), investigation (equal), methodology (equal), writing – original draft (equal), writing – review and editing (equal). **Gertrude Okiko:** formal analysis (equal), investigation (equal), methodology (equal), writing – original draft (equal), writing – review and editing (equal). **George Wanjala:** investigation (equal), methodology (equal), writing – original draft (equal), writing – review and editing (equal). **Susan Luvitaa:** investigation (equal), methodology (equal), writing – original draft (equal), writing – review and editing (equal). **Boniphase Oure:** formal analysis (equal), investigation (equal), methodology (equal), writing – original draft (equal), writing – review and editing (equal). **Frank Vriesekoop:** writing – original draft (equal), writing – review and editing (equal). **Claire D. Munialo:** conceptualization (lead), formal analysis (lead), investigation (equal), project administration (lead), supervision (lead), writing – original draft (equal), writing – review and editing (equal).

## Ethics Statement

The authors have nothing to report.

## Conflicts of Interest

The authors declare no conflicts of interest.

## Data Availability

The authors have nothing to report.
